# Genome-wide and single-base resolution DNA methylomes of the Pacific oyster *Crassostrea gigas* provide insight into the evolution of invertebrate CpG methylation

**DOI:** 10.1186/1471-2164-15-1119

**Published:** 2014-12-16

**Authors:** Xiaotong Wang, Qiye Li, Jinmin Lian, Li Li, Lijun Jin, Huimin Cai, Fei Xu, Haigang Qi, Linlin Zhang, Fucun Wu, Jie Meng, Huayong Que, Xiaodong Fang, Ximing Guo, Guofan Zhang

**Affiliations:** Institute of Oceanology, Chinese Academy of Sciences, Qingdao, China; China National GeneBank, BGI-Shenzhen, Shenzhen, China; Centre for GeoGenetics, Natural History Museum of Denmark, University of Copenhagen, Copenhagen, Denmark; BGI-Shenzhen, Shenzhen, China; Haskin Shellfish Research Laboratory, Institute of Marine and Coastal Sciences, Rutgers University, Port Norris, NJ 08349 USA

**Keywords:** Mollusca, Oyster, *Crassostrea gigas*, DNA methylation, Genome-wide, Gene age

## Abstract

**Background:**

Studies of DNA methylomes in a wide range of eukaryotes have revealed both conserved and divergent characteristics of DNA methylation among phylogenetic groups. However, data on invertebrates particularly molluscs are limited, which hinders our understanding of the evolution of DNA methylation in metazoa. The sequencing of the Pacific oyster *Crassostrea gigas* genome provides an opportunity for genome-wide profiling of DNA methylation in this model mollusc.

**Results:**

Homologous searches against the *C. gigas* genome identified functional orthologs for key genes involved in DNA methylation: *DNMT1*, *DNMT2*, *DNMT3, MBD2/3* and *UHRF1*. Whole-genome bisulfite sequencing (BS-seq) of the oyster’s mantle tissues revealed that more than 99% methylation modification was restricted to cytosines in CpG context and methylated CpGs accumulated in the bodies of genes that were moderately expressed. Young repeat elements were another major targets of CpG methylation in oysters. Comparison with other invertebrate methylomes suggested that the 5’-end bias of gene body methylation and the negative correlation between gene body methylation and gene length were the derived features probably limited to the insect lineage. Interestingly, phylostratigraphic analysis showed that CpG methylation preferentially targeted genes originating in the common ancestor of eukaryotes rather than the oldest genes originating in the common ancestor of cellular organisms.

**Conclusions:**

Comparative analysis of the oyster DNA methylomes and that of other animal species revealed that the characteristics of DNA methylation were generally conserved during invertebrate evolution, while some unique features were derived in the insect lineage. The preference of methylation modification on genes originating in the eukaryotic ancestor rather than the oldest genes is unexpected, probably implying that the emergence of methylation regulation in these 'relatively young’ genes was critical for the origin and radiation of eukaryotes.

**Electronic supplementary material:**

The online version of this article (doi:10.1186/1471-2164-15-1119) contains supplementary material, which is available to authorized users.

## Background

DNA methylation is one of the most important epigenetic modifications of the eukaryotic genomes, which is believed to play important roles in diverse biological processes, such as regulation of temporal and spatial gene expression [1], alternative splicing [2], control of transcriptional noise [3] and genome stabilization [4]. In recent years, advances in profiling methylated DNA by next generation sequencing have promoted the production of DNA methylomes on more than 20 eukaryotic organisms, including fungi, plants, invertebrates and vertebrates [5–10]. Comparison of DNA methylation patterns across such a wide range of taxa uncovers some phylogenetically conserved characteristics of eukaryotic DNA methylation (e.g. gene body methylation), but also reveals that the genome-wide degree, distribution and function of DNA methylation vary greatly among taxa [5, 6]. For example, about 4-6% of genomic cytosines are methylated in humans [7, 11], whereas only around 0.1-0.2% of cytosines are methylated in insects [8–10]; repetitive elements are heavily methylated in plants and vertebrates, but rarely in invertebrates studied so far [5, 6]; methylation around transcriptional start sites (TSS) silences transcription in vertebrates and some plants, whereas gene body methylation is generally associated with high expression level in plants, invertebrates and vertebrates [5–7, 10, 12, 13]. Characterizing the DNA methylomes in other poorly sampled taxa is essential for a better understanding of the evolution of DNA methylation as well as its functions and biological significance in eukaryotes.

Mollusca is one of the most species-rich phyla in the animal kingdom. Recently, some studies have revealed the presence and the potential importance of DNA methylation in several molluscan species [14–18], while the majority of studies so far have been focused on the Pacific oyster *C. gigas*, probably due to the abundance of EST resource [19] and the available of genome sequence for this species [20]. Oysters are bivalve molluscs widely distributed in world oceans and estuaries, as ecological keystone species, as well as important fishery and aquaculture species. As sessile species living in the intertidal zone and estuaries, oysters tolerate extremely fluctuations in temperature, salinity and air exposure, which make them excellent model species to study the molecular mechanisms (e.g. epigenetic regulation) of stress adaption [20].

The presence of DNA methylation in *C. gigas* was first reported by Gavery and Roberts [14]. Subsequently, Gavery and Roberts [17] adopted a strategy of combining methylation enrichment and BS-seq to describe the genome-wide distribution of DNA methylation in gill tissues of oyster and showed that methylated genes were generally associated with high transcript abundance. More recently, Olson and Roberts [21] characterized the DNA methylation profile in male gamete cells using whole-genome bisulfite sequencing and confirmed the previous findings of Gavery and Roberts. Interestingly, they also reported a positive association between methylation level of promoter regions and gene expression [21], against that increased methylation in promoter regions corresponds to decreased gene expression of homeobox genes in oysters reported by Riviere *et al.* [16].

Although our knowledge of DNA methylation modification in the Pacific oyster is increasing in recent years, comprehensive analyses of the characteristics and the functions of DNA methylation in this species are still lacking. In addition, DNA methylomes from other tissues are also crucial for better understanding of DNA methylation regulation in oysters. In this study, we performed unbiased whole-genome BS-seq for the mantles of two individual oysters to generate DNA methylomes at single-base resolution for this model mollusc. We then analyzed the general characteristics of DNA methylation in oysters, and compared it with published methylomes from other species to provide insight into the evolution of CpG methylation in invertebrates.

## Results and discussion

### The oyster genome encodes a complete DNA methylation toolkit

**Figure 1:**
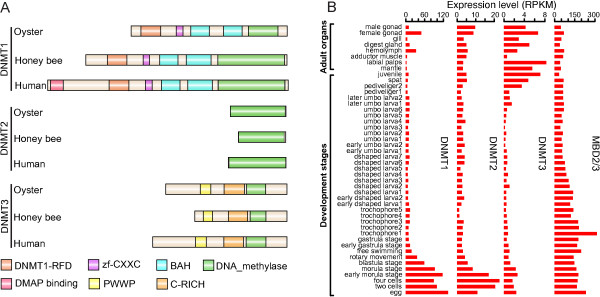
**DNA methylation related genes in the oyster genome. (A)** Conserved domain organization of the DNMT1, DNMT2 and DNMT3 proteins among oyster *Crassostrea gigas*, honeybee *Apis mellifera* and human *Homo sapiens*. Honeybees have two *DNMT1* genes (*DNMT1A* and *DNMT1B*), and the DNMT1A protein is presented. Human have three *DNMT3* genes (*DNMT3A*, *DNMT3B* and *DNMT3L*), and the DNMT3A protein is presented. **(B)** Expression levels of the oyster *DNMTs* and *MBD2/3* genes in different developmental stages and adult organs, denoted by reads per kilobase of transcript per million mapped reads (RPKM). Numbers after sample names indicate the orders in development, with smaller numbers representing earlier stages in development.

In metazoa, methylation of cytosines is catalyzed by a family of DNA methyltransferases (DNMTs) classified into three groups (DNMT1, 2 and 3), and sites of DNA methylation are recognized by a family of proteins that contain conserved methyl-CpG binding domains (MBDs) [22, 23]. Genes encoding DNMTs and MBDs constitute the genetic toolkit of DNA methylation. Gavery and Roberts [14] first reported the presence of *DNMT1*, *DNMT3* and *MBD2* genes in *C. gigas* by homologous search against the EST database of *C. gigas* (GigasDatabase), but they failed to identify the *DNMT2* homolog. By performing homologous searches against the *C. gigas* genome, we identified one ortholog each for *DNMT1*, *DNMT2*, and *DNMT3,* which share conserved domain organizations with that of other animals (Figure 1A). Besides, we found that the vertebrate *MBD2* and *MBD3* protein sequences matched to the same locus in the *C. gigas* genome, consistent with previous reports in other invertebrates that *MBD2* and *MBD3* occur as a unique gene (*MBD2/3*) in invertebrate genomes [24].

By analyzing the extensive transcriptome data produced in the *C. gigas* genome project [20], we found that the oyster *DNMTs* and *MBD2/3* were widely expressed across different developmental stages and adult organs (Figure 1B). *DNMT1* is implicated in post-replication maintenance of methylation patterns, ensuring the faithful transfer of the methylation status of parental DNA to the newly synthesized DNA strand [22, 23]. We observed that the expression levels of the oyster *DNMT1* were relatively high in eggs and at early developmental stages (Figure 1B), consistent with the requirement of methylation maintenance during early developmental stages when cell divisions are particularly active. Of note, *UHRF1* (ubiquitin-like with PHD and ring finger domains 1), an important cofactor that works with *DNMT1* to maintain CpG methylation during DNA replication in other species [6], also remained conserved in the *C. gigas* genome and showed highly similar expression pattern to that of *DNMT1* (Pearson’s *r* = 0.86, *p* < 10^-13^; Additional file 1: Figure S1), implying its conserved role in the maintenance of CpG methylation in *C. gigas*.

*MBD2* was highly expressed during early developmental and peaked at the early trochophore stage (Figure 1B), likewise supporting the important role of DNA methylation regulation in early embryonic developments of oysters. In contrast, the expression levels of *DNMT3*, which is required for *de novo* methylation in other species [22, 23], were increased in the pediveliger and latter stages (Figure 1A). In the pediveliger stage, oysters begin to crawl and search for hard substrates for settlement. Thus, pediveliger represents the beginning of sessile life style of oysters that must cope with harsh and dynamically changing environments [25]. During settlement, pediveligers go through metamorphosis and significant transformation of their body including the dissolution of velum and foot and formation of gills. The elevation of *DNMT3* expression at this stage probably indicated that the establishment of new DNA methylation patterns is essential for oysters to form a new body plan and to cope with the new sessile lifestyle and environment. Taken together, our data suggested that the oyster genome encoded a complete and functional genetic toolkit for DNA methylation and supported that DNA methylation appeared to be important for oyster’s early development [16].

### Genome-wide methylation level of oyster is much higher than insects but close to other invertebrates

To investigate the DNA methylation pattern of *C. gigas*, we performed BS-seq on bisulfite-converted DNA extracted from the mantle tissues of two different individuals: one inbred individual used for the oyster genome project [20] and one wild individual obtained from Weihai, Shandong Province, China (see Methods). We sequenced approximately 25 and 28 Gb of data for the inbred and wild individuals, respectively, which covered the two genomes at an average depth of approximately 16X (inbred) and 12X (wild) per strand after read mapping and subsequent filtering (Additional file 1: Table S1; see Methods). About 95% and 73% of genomic cytosines (Cs) were covered by at least two unique reads in the inbred and wild samples (Additional file 1: Table S2), respectively. The relatively low coverage of the wild sample is probably attributed to the high polymorphism or sequence differences between the wild and the reference (inbred) genome [20], which hindered read alignment. To estimate the non-conversion rate of our bisulfite conversion, we added unmethylated lambda DNA into each library (see Methods). On average, about 0.4% of the cytosines (Cs) were estimated to fail in C–T conversion during bisulfite treatment for each library (Additional file 1: Table S1).

We then considered the high sequencing depth of each sample and conducted a statistical model based on the binomial distribution to distinguish true methylated cytosines (mCs) from the experimental noise with a false discovery rate (FDR) under 1% [10] (see Methods). We identified 3,055,909 and 2,391,673 mCs, accounting for 1.95% and 1.96% of all the covered Cs (≥2X) in the inbred and wild genomes (Table 1), respectively. Of note, these genome-wide mC ratios (~2%) are an order of magnitude higher than that observed in insects, such as silkworm (0.11%), honeybee (0.11%) and ant (0.15%), but close to that of sea anemone (1.44%), sea squirt (4.07%) and human (3.93%) (Table 1), regardless of the relatively closer phylogenetic relationship between oyster and insects than other species used for comparison in this study. Among all the mCs, more than 99% were in the CpG context, consistent with that observed in other animal methylomes (Table 1). Most of the methylated CpGs (mCGs) exhibited high methylation levels (Additional file 1: Figure S2). If only considering CpG methylation, we could observe that up to 16% of the covered CpGs (≥2X) were modified by DNA methylation in the inbred and wild mantle genomes, close to the previous estimation of 15% in the gill tissues and male gametes in the same species [17, 21], and also close to that of sea anemone (11%) and sea squirt (29%), but much higher than that observed in insects (0.36-0.56%) (Table 1).

**Table 1:** **General characteristics of DNA methylation in different species**

Species	Tissue	% of mC in genomic cytosines	% of mCG in genomic CpGs	% of mC in different sequence context			% of genes being methylated	Level of gene body methylation	Data sources
				CG	CHG	CHH			
Inbred oyster	Mantle	1.95	15.96	99.56	0.08	0.36	54	0.20	N/A
Wild oyster	Mantle	1.96	16.25	99.69	0.06	0.25	58	0.18	N/A
Silkworm	Silk gland	0.11	0.56	99.24	0.14	0.62	47	0.02	[8]
Honeybee	Adult brain	0.11	0.36	97.36	0.10	2.54	61	0.01	[9]
Ant	Embryo	0.15	0.54	99.25	0.03	0.72	31	0.02	[10]
Sea anemone	Whole adult	1.44	10.89	99.93	0.02	0.05	51	0.16	[5]
Sea squirt	Muscle	4.07	28.95	99.94	0.01	0.05	71	0.34	[5]
Human	Peripheral blood	3.93	82.08	92.40	0.70	6.90	97	0.68	[11]

Note: For sequence context of CHG or CHH, H represents A, T or C. When calculating the percentage of mC out of total genomic cytosines or mCGs out of total genomic CpGs, only C or CpG positions with ≥ 2X coverage were considered. Methylated genes were defined as genes with ≥ 2 identified mCGs. Only genes with ≥ 70% coverage were used for gene-related calculation, and only CpG positions with ≥ 5X coverage were used for gene body methylation level estimation. Species used for comparison are the oyster *Crassostrea gigas*, silkworm *Bombyx mori*, honeybee *Apis mellifera*, ant *Camponotus floridanus*, sea anemone *Nematostella vectensis*, sea squirt *Ciona intestinalis* and human *Homo sapiens*.

### Accumulation of mCGs in gene bodies and repetitive elements

**Figure 2:**
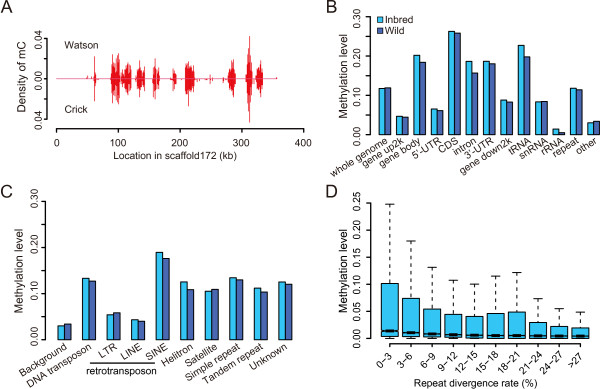
**Genomic distribution of mCGs. (A)** Density of mCG across scaffold172 in the inbred sample. A 1-kb non-overlapping sliding window was used for density estimation, which was calculated as the number of identified mCGs divided by the total length of a window. **(B)** Methylation levels of different genomic elements. The 'gene body’ category includes untranslated regions (5’-UTR and 3’-UTR, i.e. non-coding exonic regions), coding sequences (CDS, i.e. coding exonic regions) and introns, and the 'other’ category represents all genomic regions excluding gene bodies, 2-kb up/downstream of gene bodies, repeats, tRNAs, snRNAs and rRNAs. **(C)** Methylation levels of different repeat classes. 'Background’ corresponds to the 'other’ category in panel **B**. **(D)** Methylation levels of repeat loci with different divergence rates. The repeat divergence rate was calculated between the identified repetitive elements in the oyster genome and the consensus sequences in the repeat library [20].

Invertebrate genomes generally display mosaic methylation patterns with methylated regions interspersed among large regions without methylation [26]. We observed that the oyster genome followed a similar mosaic methylation pattern (Figure 2A).

To investigate the potential regulatory targets of DNA methylation in oyster, we estimated the methylation level of different genomic regions. As methylation modification is exclusively restricted to cytosines in CpG context (Table 1), we decided to focus solely on CpG methylation for subsequent analyses. We first calculated the methylation level of an individual CpG by dividing the number of reads containing a C at the site of interest divided by the total number of reads covering the site. Then, the methylation level of a specific region was determined by the sum of methylation levels of individual CpGs in the region divided by the total number of covered CpGs in this region. Only CpG positions with ≥ 5X coverage were considered in methylation level calculation. Our data revealed that CpGs of the protein-coding genes, particularly their coding exons (CDSs), were most frequently subjected to methylation modification compared with other genomic elements in the oyster genome (Figure 2B), a pattern similar to that found in other invertebrates [5, 6, 8, 10]. For the three classes of non-coding RNA loci investigated here, CpG methylation also frequently targeted transfer RNA (tRNA) loci, whereas in a less extent for small nuclear RNAs (snRNA) and relatively rare for ribosomal RNAs (rRNA) (Figure 2B).

While repetitive elements are generally heavily methylated in plants and vertebrates, they are reported to be rarely methylated in insects or sea anemone and moderately methylated in sea squirt [5, 6, 8, 10]. The overall methylation level of repetitive elements in *C. gigas* was twice higher than the genome background (i.e. intergenic regions with no annotations) (Figures 2C), implying that there should be a fraction of repetitive loci targeted by CpG methylation. When dividing repetitive elements into different classes, we observed that the methylation levels of long interspersed elements (LINEs) and long terminal repeats (LTRs) were close that of the genome background, indicating the depletion of CpG methylation in these two kind of retrotransposons. In contrast, another major class of retrotransposon, short interspersed elements (SINEs), displayed the highest methylation level among all the repeat classes, close to that of genic regions (Figure 2B-C). In addition, other repeat classes, such as DNA transposons, helitrons, satellites, simple repeats and tandem repeats, all displayed methylation levels twice higher than the genome background (Figure 2C). These results indicated that DNA methylation in the oyster genome only targeted some particular classes of repetitive elements, in sharp contrast to the observation in vertebrate genomes [5–7, 11].

Furthermore, we observed a general negative relationship between repeat methylation level and repeat divergence rate (Inbred: Spearman’s *r* = -0.15 and *p* < 10^-15^; Wild: *r* = -0.16 and *p* < 10^-15^; Figure 2D). In addition, if we divided the repeats into methylated (i.e. repeats targeted by at least two identified mCGs) and unmehylated groups, we actually observed that the divergence rate of the methylated group was significantly lower than that of the unmethylated group (Wilcoxon rank-sum test *p* < 10^-15^; Additional file 1: Figure S3), suggesting that DNA methylation preferentially target young repetitive elements, which are more likely to be active in the oyster genome.

### Gene body methylation, gene length and gene expression

**Figure 3:**
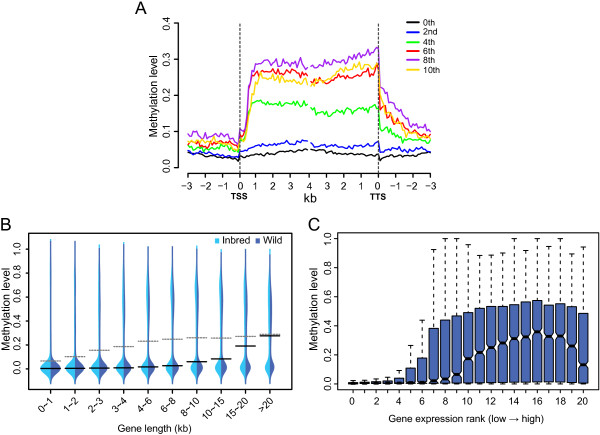
**Gene body methylation, gene length and gene expression. (A)** Methylation levels across the bodies of genes in different expression ranks. The 0^th^ rank represents all silent genes (RPKM = 0), while the expressed genes (RPKM > 0) were binned from 1 (least expressed) to 10 (most expressed) ranks. Genes were aligned at the transcription start sites (TSS, left dashed line) or the transcription termination sites (TTS, right dashed line), and average methylation levels for each 100-bp interval are plotted. **(B)** Distribution of gene body methylation levels across genes in different length intervals. Solid black and gray dashed lines denote the median and mean methylation levels for each group, respectively. Width of each shape at a given y value shows the relative frequency of genes present in that methylation level. **(C)** Distribution of gene body methylation levels across genes in different expression ranks. Genes were ranked as described in panel **A**, but expressed genes were divided into 20 bins.

We then profiled the DNA methylation levels across gene bodies and found that DNA methylation level increased sharply after the transcription start sites (TSS), remained a plateau along the gene bodies and slowly dropped back to the genome background levels after the transcription termination sites (TTS) (Figure 3A). This pattern is similar to that observed in sea squirt [5], but quite different from that in insects, where mCGs were particularly accumulated in the 5’ end, especially the second exons of genes [5, 10]. In addition, although the fraction of genes with methylation modification (i.e. genes targeted by at least two identified mCGs) were similar among oyster, sea anemone, sea squirt and insects (Table 1), the overall methylation levels of gene bodies in oyster (~0.19) were an order of magnitude higher than that observed in insects (0.01-0.02), but close to that of sea anemone (0.16) and sea squirt (0.34) (Table 1).

It has been reported that genes with high methylation levels are significantly longer than that with low methylation levels in sea anemone and sea squirt, but the opposite results are observed in silkworm, honeybee and ants [10, 27]. In oyster, we observed that the gene body methylation levels had a generally positive correlation with the gene lengths (Inbred: Spearman’s *r* = 0.36 and *p* < 10^-15^; Wild: *r* = 0.39 and *p* < 10^-15^; Figure 3B). Consistently, if we divided the genes into methylated (i.e. genes targeted by at least two identified mCGs) and unmehylated groups, we observed that methylated genes were significantly longer than that of unmethylated genes (Wilcoxon rank-sum test *p* < 10^-15^; Additional file 1: Figure S4). Thus, our observation in oyster was consistent with that observed in sea anemone and sea squirt and supporting that the gene body methylation pattern of insects might represent an exception during the evolution of gene body methylation in invertebrates.

Gene body methylation is considered as an important mechanism in the regulation of gene expression [1, 5, 6, 12]. By analyzing the RNA-seq data from the mantle tissues of the same wild individual used for methylation analysis [20], we found that gene body methylation levels were generally positively correlated with gene expression levels except the most highly expressed genes (Figure 3A and C). This was also supported by correlation analysis that the Spearman's *r* between gene body methylation and expression level was 0.46 (*p* < 10^-15^) for all genes but was raised to 0.50 (*p* < 10^-15^) after excluding the top 20% most highly expressed genes. Our result was consistent with previous analyses of gill and male gametes of *C. gigas* [17, 21], and also consistent with observations in plants and other invertebrates where moderately expressed genes have higher methylation levels than lowly or highly transcribed ones [5, 6, 8, 10, 12], indicating the conserved role of gene body methylation on gene expression regulation. Of note, a negative correlation between gene expression and promoter methylation level was reported in some oyster development related genes [16]. However, we could not observed this negative correlation at the genome level (Figure 3A), agreeing with a recent report in oyster male gametes [21] and suggesting that suppression of gene expression by promoter methylation is not a general mechanism in oyster, probably only operating on a small proportion of genes.

### CpG methylation preferentially targets genes originating in the ancestor of eukaryotes rather than the oldest genes

**Figure 4:**
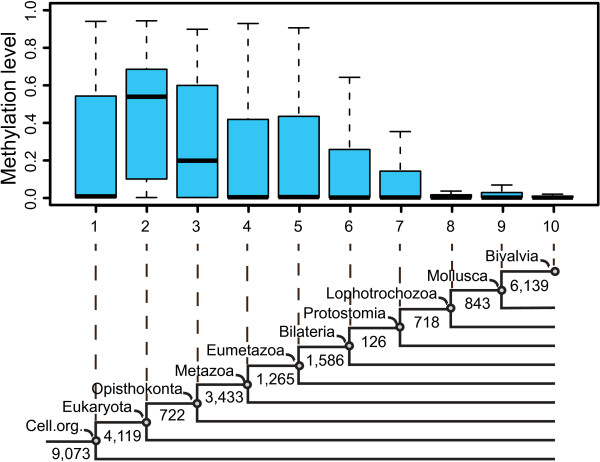
**Gene body methylation level across gene age.** All the oyster genes were first mapped to different phylogenic nodes by the phylostratigraphic analysis, with 1 representing the oldest and 10 representing the youngest groups, respectively. Then the methylation levels for genes in different age groups were visualized by boxplot. The lower part of the panel depicts the 10 phylostrata used in this analysis, with the numbers of oyster genes mapped in the different phylostrata indicated.

Previous studies have uncovered that CpG methylation preferentially target housekeeping genes that are constitutively expressed, evolutionarily conserved and encoding essential cellular functions [1, 8, 10, 27, 28]. Genes that have been preserved for the longest time during evolution should be the most likely to take part in housekeeping functions. Thus, one may expect that CpG methylation preferentially targets the oldest genes that originated the earliest in evolution. To investigate the CpG methylation pattern across genes with different evolutionary origins, we performed a phylostratigraphic analysis [29] and placed all oyster genes into 10 phylostrata, which represented different evolutionary origins (or ages) of the genes (Figure 4).

Interestingly, we observed that the oldest genes, which could be dated back to the last common ancestor (LCA) of cellular organisms (phylostratum 1), did not show the highest methylation levels (Figure 4). In contrast, methylation levels peaked in the genes originating in the LCA of eukaryotes (phylostratum 2), and subsequently declined with gene age, with the lineage-specific genes originating after Protostomia (phylostratum 8-10) showing the lowest methylation levels. This divergent pattern of CpG methylation for genes with different origins is in fact conserved in insects, sea anemone and sea squirt (Additional file 1: Figure S5), indicating that it is a common characteristic in metazoa.

Gene ontology (GO) enrichment analysis for genes from different phylostrata revealed that genes originating in LCA of cellular organisms (phylostratum 1) were significantly over-represented in the housekeeping biological processes of small molecule metabolism (e.g. ketone, amino acid, nitrogen compound, alcohol and tetrapyrrole), oxidation-reduction reaction and cellular component biogenesis (Additional file 2: Table S3) whereas genes originating in LCA of eukaryotes (phylostratum 2) were particularly enriched in other kinds of housekeeping functions, such as macromolecule or cellular localization, vesicle-mediated transport, cell cycle, microtubule-based processes and macromolecule metabolic processes (Additional file 2: Table S4), confirming that CpG methylation tend to target some specific classes of housekeeping genes with particular functions. The preferential targeting of genes originating in the ancestor of eukaryotes rather than the oldest genes also implies that the emergence of novel genes together with the acquirement of a more complex regulatory mechanism by DNA methylation on these genes might be involved in the origin and later radiation of eukaryotes.

## Conclusions

Our study presented two genome-wide DNA methylomes at single-base resolution for the mantle tissues of the Pacific oyster *C. gigas*, and provided a comprehensive investigation of the characteristics of DNA methylation in this species. We uncovered that the oyster genome preserved a complete and functional genetic toolkit for DNA methylation, encoding conserved enzymes that are capable of both maintenance and *de novo* methylation and MBD proteins that may bind to methylated cytosines. By comparing with the methylomes of several insects, a sea anemone and a sea squirt, we observed several conserved properties of DNA methylation in oysters, including the predominance of DNA methylation modification in CpG dinucleotide context, the accumulation of mCGs in gene bodies especially their coding exons, the high methylation levels for moderately expressed genes with housekeeping functions and the general lack of promoter methylation. We also provide evidences for the selective methylation of some young repetitive elements, particularly SINEs in the oyster genome.

Previous studies reveal that insects show some unique characteristics of DNA methylation when compared with sea anemone (Cnidaria) and sea squirt (Chordata), such as the extremely low genome-wide methylation levels with only 0.1-0.2% genomic cytosines being methylated, a strong bias of CpG methylation towards 5’-end of gene bodies and the negative correlation between gene body methylation level and gene length [5, 8–10, 27], although the origin of these derived features during evolution remains poorly understood. Oysters and insects are both protostomes and relatively close-related in phylogeny among invertebrates with published methylomes so far. Thus, the generally more similar methylation patterns between oyster and sea anemone/sea squirt than with insects suggest that the insect-like methylation pattern may not be derived from the ancestor of protostomes. Of note, recent insight from the examination of CpG depletion in the termite genome reveals that the genome-wide methylation level in termites (hemimetabolous) is higher than that in hymenopteran insects (e.g. honeybees and ants, holometabolous), and CpG methylation seems to target the entire length of gene bodies instead of bias toward the 5’-end [30], implying that the above insect-like methylation patterns are probably derived during the evolution of holometabolous insects.

Finally, the relatively higher methylation levels observed for the evolutionarily older genes support the role of CpG metylation in genes performing housekeeping functions. However, the preferential targeting of genes originating in the ancestor of eukaryotes rather than the oldest genes is unexpected. Gene body methylation is proposed to be associated with regulating transcript abundance and alternative splicing [2, 9, 10, 12, 31, 32], which may provide important foundations for organismal adaptation. Thus, our results likely imply that the emergence of novel genes in the ancestor of eukaryotes together with the acquirement of a more complex regulatory mechanism by CpG methylation on these genes was important in the origin and later radiation of eukaryotes.

## Methods

### Homologous search of *DNMT*, *MBD*and *UHRF1*genes in the oyster genome

DNMT, MBD and UHRF1 protein sequences of *Homo sapiens*, *Danio rerio*, *Takifugu rubripes*, *Gallus gallus*, *Ciona intestinalis* and *Apis mellifera* collected from NCBI were used as queries for homologous searches in the oyster genome. We first mapped the query proteins to the oyster genome with TBLASTN [33] (E-value ≤ 0.01), and excised target-gene regions with 2-kb flanking sequences for gene structure and protein sequence determination using GeneWise [34]. Then, we aligned the predicted proteins to the NCBI NR database to check whether the predicted proteins were expected genes. Domain organization of the DNMT proteins was predicted using Pfam [35] (http://pfam.sanger.ac.uk) with default settings.

### Gene expression analysis

Transcriptome data of different developmental stages and adult organs were from the oyster genome project [20], which have been deposited in the Gene Expression Omnibus under the accession number GSE31012. TopHat v2.0.4 package [36] was employed to map transcriptome reads to the oyster genome with default settings. Gene expression levels were measured by RPKM (Reads Per Kilobase of transcript per Million mapped reads) [37], and adjusted by a scaling normalization method [38].

### Biological material and DNA extraction for methylome analysis

Two Pacific oysters were used for methylome profiling in this study. One is an inbred oyster (05x7-T-G4-1.051#20) that was produced by four generations of sister-brother mating (coefficient of inbreeding, *F* = 0.59) and has been used for whole genome-sequencing [20]. The other was a wild oyster collected from Weihai, Shandong Province, China. Both oysters were about two years of age. The inbred oyster was produced as single oysters and cultured intertidally at southern Puget Sound, Washington, USA, where the water temperature ranges from 7 to 16°C. The wild oyster was an attached oyster from an oyster farm in its native range at Weihai, China, where the water temperature ranges from 4 to 27°C. Genomic DNA of the mantle tissues was extracted as described previously [20].

### BS-seq library construction and sequencing

For DNA from each of the two individuals, we constructed two independent libraries. For each library, 5 μg genomic DNA mixed with 25 ng cl857 Sam7 Lambda DNA was fragmented by sonication with a Covaris S2 system (Covaris, MA) to a mean size of approximately 250 bp. End-repair, 3ʹ-end dA addition and adapter ligation were subsequently performed. Methylated adapters were used according to the manufacturer’s instructions (Illumina). The bisulfite conversion of DNA was performed according to a modified NH4HSO3-based protocol [39] and amplified with nine cycles of PCR. All libraries were subjected to 90-bp paired-end sequencing on an Illumina HiSeq 2000 platform.

### BS-seq analysis

The Lambda genome was added into the reference genome of *C. gigas* (oyster.v9.fa.gz from http://gigadb.org/dataset/100030) so that reads originating from the unmethylated control DNA could be aligned. BS-seq reads were mapped to the reference genome using SOAP2 [40] as described in [10], allowing no more than 4 mismatches for 90 bp reads. Multiple reads mapping to the same position were regarded as PCR duplicates, and only one of them was kept. Bases with a quality score < 30 were not considered for subsequent analysis.

The error rate of each library (sum of the non conversion rate and T/C sequencing errors) was calculated as the total number of sequenced Cs divided by the total sequencing depth for sites corresponding to Cs in the Lambda genome. The error rate for each library was ~0.4% (Additional file 1: Table S1). To distinguish true mCs from false positives, we used a model based on the binomial distribution following [10], and only the mCs with FDR [41] adjusted *P*-values < 0.01 were considered true positives.

### Methylation level calculation

Methylation level of an individual CpG was determined by the number of reads containing a C at the site of interest divided by the total number of reads covering the site. Methylation level of a specific region was determined by the sum of methylation levels of individual CpGs in the region divided by the total number of covered CpGs in this region. Only cytosine positions covered by at least 5 unique reads were used in methylation level analysis.

### Phylostratigraphic analysis

Evolutionary origin (age) of the oyster genes were determined by a phylostratigraphic analysis [29], and finally divided into 10 phylostrata ranging from (1 to 10) cellular organism, Eukaryota, Opisthokonts, Metazoa, Eumetazoa, Bilateria, Protostomia, Lophotrochozoa, Mollusca, and Bivalvia. The analysis procedure is done in two steps. First, a consensus phylogeny is created, in which each node is represented by one or more fully sequenced genomes. Then, the origin of the genes from an extant genome (oyster in this analysis) is mapped to a particular node in this phylogeny (called phylostratum) based on the BLASTP analysis.

Following Domazet-Lošo T *et al.* [42], we chose NCBI NR database for the phylostratigraphic analysis as it represents the most exhaustive set of known proteins across all organisms. Before the sequence similarity search, the NR database was cleaned up with respect to sequences with uncertain taxonomic status (for example, those annotated as 'incerteae sedis’ , 'environmental samples’ or 'unclassified’) or where the taxonomy ID is not included in the cellular organisms section of the NCBI taxonomy database as described by Domazet-Lošo T *et al.* [42]. In addition, we removed sequences of metazoan taxa with currently unreliable phylogenetic position (Mesozoa, Myxozoa, Chaetognatha and Placozoa) from the database. The final species used in this analysis are listed in Additional file 2: Table S5. Then, we mapped the oyster proteins to the cleaned-up NR database using BLASTP [33] (E-value < 0.001) and determined the origin of each oyster gene based on the phylogenic node annotation of the hit gene.

Fisher’s exact test and χ^2^ test were employed to estimate whether the genes from a given phylostratum were enriched in specific GO categories when compared with background genes [43]. *P*-values were adjusted by FDR [41], and the adjusted *P*-value < 0.05 was chosen as the significant threshold.

### Availability of supporting data

Sequencing data generated for this study have been deposited in the NCBI GEO database as GSE40302. This comprises the subseries GSM991064 (Inbred), and GSM991065 (Wild).

## Electronic supplementary material

Additional file 1: Figure S1: Domain organization and expression pattern of *UHRF1* in *C. gigas*. **Figure S2.** Distribution of the methylation levels of mCs in different sequence contexts (H = A, T or C). **Figure S3.** Comparison of sequence divergence rate between unmehylated and methylated repeats. **Figure S4.** Comparison of gene length between unmehylated and methylated genes. **Figure S5.** Gene body methylation level across gene age in different invertebrates. **Table S1.** Statistics of BS-Seq for each sample. **Table S2.** Ratio of cytosines covered by at least two unique reads in different sequence contexts. H represents A, T, or C. (PDF 337 KB)

Additional file 2: Table S3: GO enrichment for genes originating in LCA of cellular organisms (i.e. genes from phylostratum 1 of figure four). **Table S4.** GO enrichment for genes originating in LCA of eukaryotes (i.e. genes from phylostratum 2 of figure four). **Table S5.** Species used in the phylostratigraphic analysis. (XLSX 186 KB)
