# Supplementary material for: Genome-wide and single-base resolution DNA methylomes of the Pacific oyster Crassostrea gigas provide insight into the evolution of invertebrate CpG methylation
Source: BMC Genomics. 2014 Dec 16;15(1):1119. doi: 10.1186/1471-2164-15-1119 (PMC4378273; doi:10.1186/1471-2164-15-1119)
Supplement: Supplementary file 1 — Additional file 1: Figure S1: Domain organization and expression pattern of UHRF1 in C. gigas. Figure S2. Distribution of the methylation levels of mCs in different sequence contexts (H = A, T or C). Figure S3. Comparison of sequence divergence rate between unmehylated and methylated repeats. Figure S4. Comparison of gene length between unmehylated and methylated genes. Figure S5. Gene body methylation level across gene age in different invertebrates. Table S1. Statistics of BS-Seq for each sample. Table S2. Ratio of cytosines covered by at least two unique reads in different sequence contexts. H represents A, T, or C. (PDF 337 KB) [file 12864_2014_6875_MOESM1_ESM.pdf]

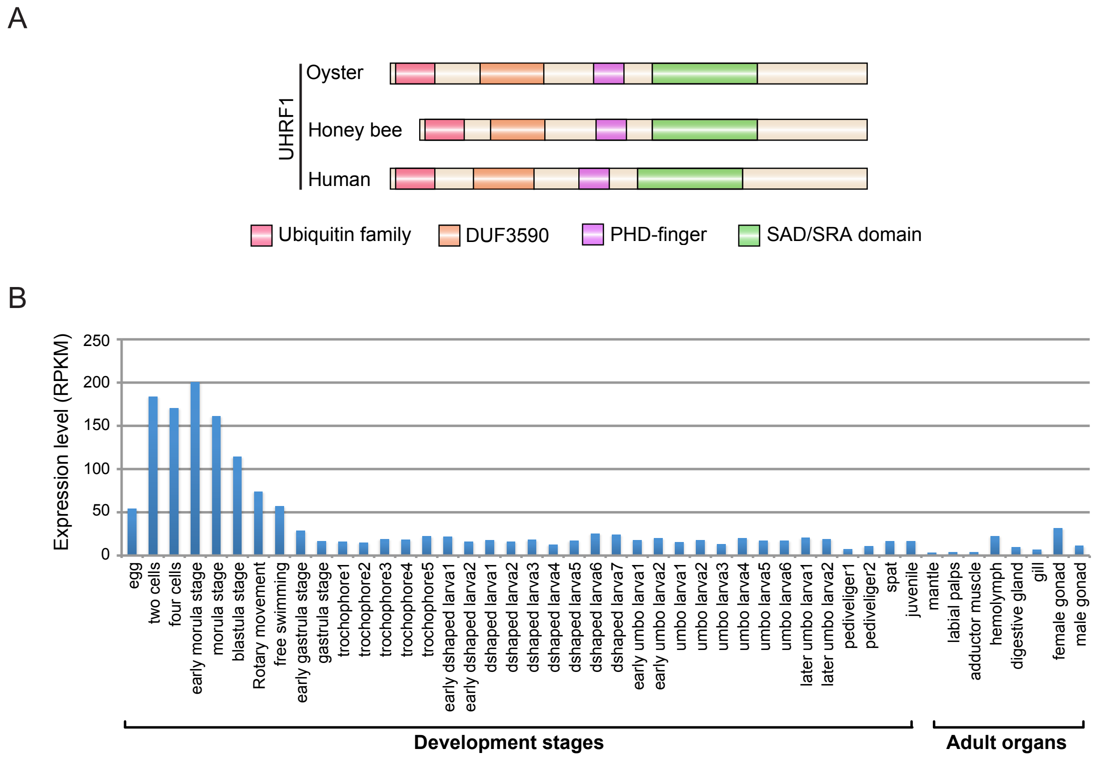

Figure S1. Domain organization and expression pattern of *UHRF1* in *C. gigas*. (**A**) Conserved domain organization of the UHRF1 protein sequences among oyster *Crassostrea gigas*, honeybee *Apis mellifera* and human *Homo sapiens*. (**B**) Expression levels of the oyster *UHRF1* gene in different developmental stages and adult organs, denoted by reads per kilobase of transcript per million reads mapped (RPKM). Numbers after sample names indicated the orders in development, with smaller numbers represented earlier stages in development.

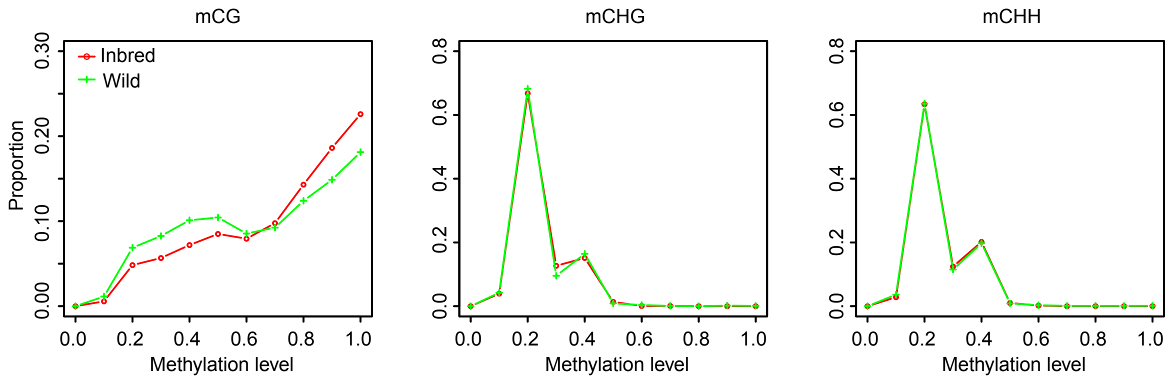

Figure S2. Distribution of the methylation levels of mCs in different sequence contexts (H=A, T or C). Methylation level for each mC is calculated by the number of reads containing a C at the site divided by the total number of reads covering the site. Only the cytosine positions covered by at least 5 unique reads were used for this analysis.

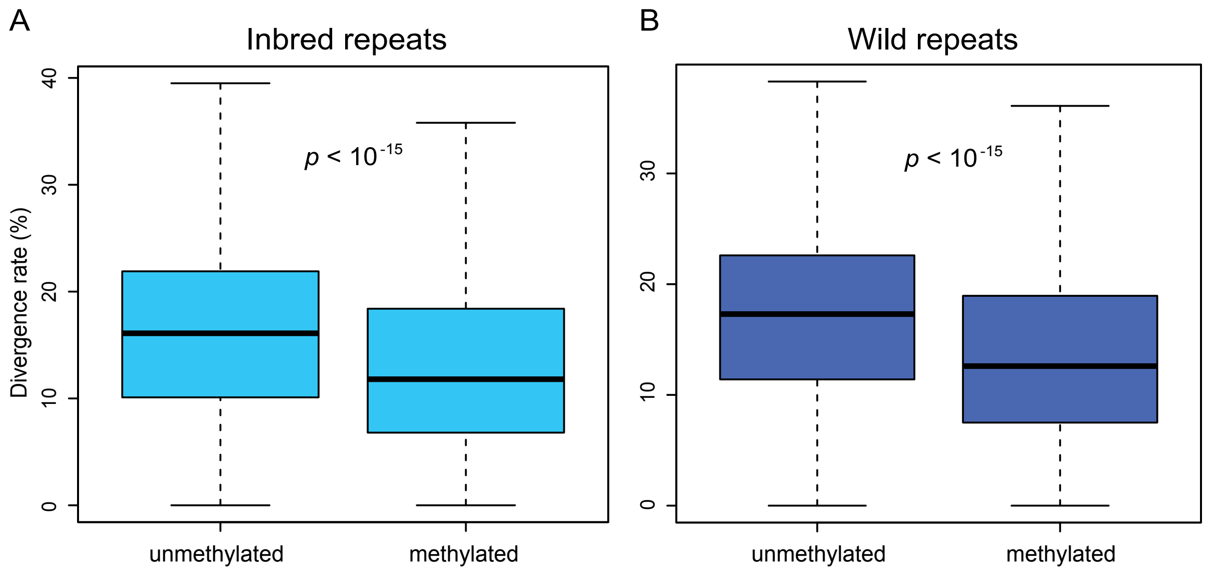

Figure S3. Comparison of sequence divergence rate between unmehylated and methylated repeats. Methylated repeats were defined as repeats targeted by at least two identified mCGs. *P*-vaules were calculated by two-sided Wilcoxon rank-sum test with a null hypothesis of no difference between two groups.

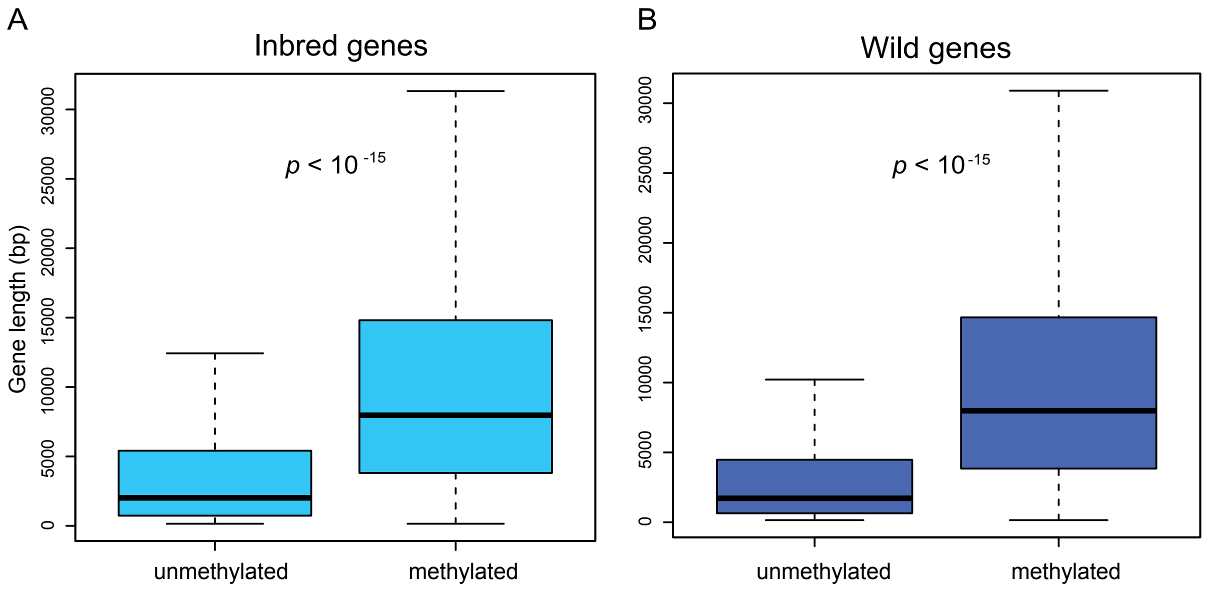

Figure S4. Comparison of gene length between unmehylated and methylated genes. Methylated genes were defined as genes targeted by at least two identified mCGs. *P*-vaules were calculated by two-sided Wilcoxon rank-sum test with a null hypothesis of no difference between two groups.

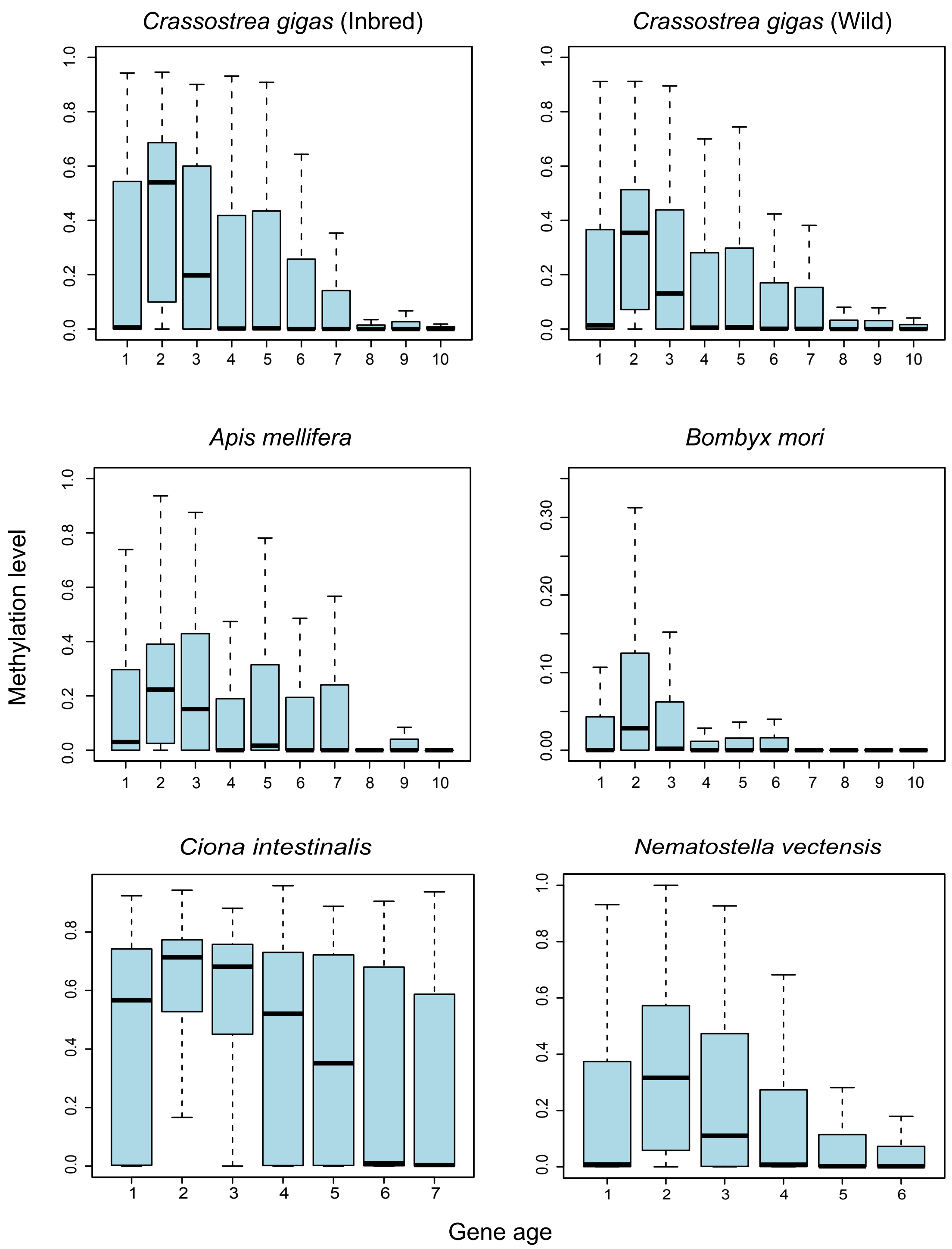

Figure S5. Gene body methylation level across gene age in different invertebrates. All the numbers in the x axis of each species are corresponding to the phylostrata described in figure 4 in the main text.

Table S1. Statistics of BS-Seq for each sample.

Sample	Library	No. of raw reads	No. of mapped reads after filtering PCR duplication	Mapped ratio (%)	No. of uniquely mapped reads	Unique mapped ratio (%)	Usable depth (X/strand)	Error rate (%)
Inbred	COSymsHAJDEABPEM	141,599,382	94,247,091	66.56	85,898,108	91.14	7.80	0.37
	COSymsHAJDEAAPEM	136,730,630	94,611,742	69.20	87,202,250	92.17	7.86	0.38
Sum		278,330,012	188,858,833	67.85	173,100,358	91.66	15.66	0.38
Wild	COSymsHADDEABPEM	157,652,694	76,331,129	48.42	65,065,037	85.24	5.89	0.39
	COSymsHADDEAAPEM	154,427,712	76,124,705	49.29	65,422,036	85.94	5.90	0.40
Sum		312,080,406	152,455,834	48.85	130,487,073	85.59	11.79	0.39

Table S2. Ratio of cytosines covered by at least two unique reads in different sequence contexts. H represents A, T, or C.

**Sample**	**Covered cytosines (%)**			
	**C**	**CG**	**CHG**	**CHH**
Inbred	94.98	95.55	95.65	94.74
Wild	73.87	73.5	75.94	73.47
